# Underreporting of Hepatitis B and C virus infections — Pennsylvania, 2001–2015

**DOI:** 10.1371/journal.pone.0217455

**Published:** 2019-06-06

**Authors:** Henry Roberts, Sameh W. Boktor, Kirsten Waller, Zahra S. Daar, Joseph A. Boscarino, Perry H. Dubin, Anil Suryaprasad, Anne C. Moorman

**Affiliations:** 1 Division of Viral Hepatitis, Centers for Disease Control and Prevention, National Center for HIV/AIDS, Viral Hepatitis, STD and TB Prevention, Atlanta, Georgia, United States of America; 2 Pennsylvania Department of Health, Harrisburg, Pennsylvania, United States of America; 3 Geisinger Health System, Danville, Pennsylvania, United States of America; 4 Hospital of the University of Pennsylvania, Department of Medicine, Philadelphia, Pennsylvania, United States of America; 5 Mayo Clinic Radiation and Oncology, Jacksonville, Florida, United States of America; FIOCRUZ, BRAZIL

## Abstract

**Context:**

In Pennsylvania, reporting of viral hepatitis B (HBV) and viral hepatitis C (HCV) infections to CDC has been mandated since 2002. Underreporting of HBV and HCV infections has long been identified as a problem. Few reports have described the accuracy of state surveillance case registries for recording clinically-confirmed cases of HBV and HCV infections, or the characteristics of populations associated with lower rates of reporting.

**Objective:**

The primary objective of the current study is to estimate the proportion of HBV and HCV infections that went unreported to the Pennsylvania Department of Health (PDoH), among patients in the Geisinger Health System of Pennsylvania. As a secondary objective, we study the association between underreporting of HBV and HCV infections to PDoH, and the select patient characteristics of interest: sex, age group, race/ethnicity, rural status, and year of initial diagnosis.

**Design:**

Per medical record review, the study population was limited to Geisinger Health System patients, residing in Pennsylvania, who were diagnosed with a chronic HBV and/or HCV infection, between 2001 and 2015. Geisinger Health System patient medical records were matched to surveillance records of confirmed cases reported to the Pennsylvania Department of Health (PDoH). To quantify the extent that underreporting occurred among the Geisinger Health System study participants, we calculated the proportion of study participants that were not reported to PDoH as confirmed cases of HBV or HCV infections. An analysis of adjusted prevalence ratio estimates was conducted to study the association between underreporting of HBV and HCV infections to PDoH, and the select patient characteristics of interest.

**Results:**

Geisinger Health System patients living with HBV were reported to PDoH 88.4% (152 of 172) of the time; patients living with HCV were reported to PDoH 94.6% (2,257 of 2,386) of the time; and patients who were co-infected with both viruses were reported to PDoH 72.0% (18 of 25) of the time. Patients living with HCV had an increased likelihood of being reported if they were: less than or equal to age 30 vs ages 65+ {PR = 1.2, [95%CI, (1.1, 1.3)]}, and if they received their initial diagnosis of HCV during the 2010–2015 time period vs the 1990–1999 time period {PR = 1.08, [95%CI, (1.05, 1.12)]}.

**Conclusion:**

The findings in this study are promising, and suggests that PDoH has largely been successful with tracking and monitoring viral hepatitis B and C infections, among persons that were tested for HBV and/or HCV. Additional efforts should be placed on decreasing underreporting rates of HCV infections among seniors (ages 65 and over), and persons who are co-infected with HBV and HCV.

## Introduction

The estimated 3.5 million hepatitis C (HCV) infections and 1.0–2.0 million hepatitis B (HBV) infections represent the largest infectious disease epidemic in the United States, but have remained largely a “silent epidemic”[1] despite their high burden of disease [2, 3, 4]. Viral hepatitis B and C infections are often characterized as silent epidemics, to some extent, because the majority of the persons infected with HBV or HCV are not aware that they are infected until they are screened for the virus, or until their liver disease progresses to cirrhosis, or hepatocellular carcinoma (HCC), several years later. Together, HBV and HCV infections are the leading causes of death from cirrhosis and HCC [1]. The high burden of HBV infections in the United States, is largely due to the migration of foreign-born persons from HBV endemic countries. Persons living with HBV in the United States are largely foreign born and may account for as many as 50% - 70% of HBV infections in the United States [3, 5]. In spite of routine childhood vaccination and targeted vaccination of at-risk populations, the prevalence of HBV infections in the United States has not seen a significant decrease in the 21^st^ century [3]. The high burden of HCV infections in the United States, in part, is due to the increasing rates of injection drug use among young adults who reside in suburban and rural communities. Recent advances in the treatment of HBV and HCV infections has fostered hope that these infections can eventually be eliminated from the US population.

Both viruses can cause either acute infection or chronic infection, a distinction that is important to both clinicians and public health officials. Acute HBV and HCV infections, are short term illnesses that occurs within six months of being exposed to the HBV and HCV, respectively. A large percentage of persons (75% - 85%) infected with HCV, will not clear the virus, and will develop a chronic HCV infection [6, 7]. A small percentage (5%) of persons infected with HBV, will not clear the virus, and will develop a chronic HBV infection [8]. Although chronic HBV and HCV infections account for 99% of all viral hepatitis infections in the United States, chronic HBV is only reportable in 46 states and chronic HCV in only 41 states [9]. Conversely, acute (new) HBV and HCV infections that meet the CDC/CSTE case definitions are reportable conditions in every state. The formal case definitions for acute and chronic HBV/HCV infections are shown in Boxes 1–4 [10].

**Box 1. CDC/CSTE Acute Hepatitis B Case Definition**

Clinical Criteria for Diagnosis

An acute illness with a discrete onset of any sign or symptom* consistent with acute viral hepatitis (e.g., fever, headache, malaise, anorexia, nausea, vomiting, diarrhea, and abdominal pain), and either a) jaundice, or b) elevated serum alanine aminotransferase (ALT) levels >100 IU/L.

Laboratory Criteria for Diagnosis

- HBsAg positive, AND
- Immunoglobulin M (IgM) antibody to hepatitis B core antigen (IgM anti-HBc) positive (if done)

Confirmed Case Classification

A case that meets the clinical case definition, is laboratory confirmed, and is not known to have chronic hepatitis B.

SOURCE: CDC/CSTE 2012 Case Definition for Acute HBV Infect

*A documented negative hepatitis B surface antigen (HBsAg) laboratory test result within 6 months prior to a positive test (either HBsAg, hepatitis B "e" antigen (HBeAg), or hepatitis B virus nucleic acid testing (HBV NAT) including genotype) result does not require an acute clinical presentation to meet the surveillance case definition.

**Box 2. CDC/CSTE Acute Hepatitis C Case Definition**

Clinical Criteria for Diagnosis

An acute illness with a discrete onset of any sign or symptom* consistent with acute viral hepatitis (e.g., fever, headache, malaise, anorexia, nausea, vomiting, diarrhea, and abdominal pain), and either a) jaundice, or b) elevated serum alanine aminotransferase (ALT) levels > 400 IU/L.

Laboratory Criteria for Diagnosis

One or more of the following three criteria:

- Antibodies to hepatitis C virus (anti-HCV) screening-test-positive with a signal to cut-off ratio predictive of a true positive as determined for the particular assay as defined by CDC. (URL for the signal to cut-off ratios: https://www.cdc.gov/hepatitis/HCV/LabTesting.htm), OR
- Hepatitis C Virus Recombinant Immunoblot Assay (HCV RIBA) positive, OR
- Nucleic Acid Test (NAT) for HCV RNA positive (including qualitative, quantitative or genotype testing)

AND, if done meets the following two criteria:

- Absence of IgM antibody to hepatitis A virus (if done) (IgM anti-HAV), AND
- Absence of IgM antibody to hepatitis B core antigen (if done) (IgM anti-HBc)

Confirmed Case Classification

A case that meets the clinical case definition, is laboratory confirmed, and is not known to have chronic hepatitis C.

SOURCE: CDC/CSTE 2012 Case Definition for Acute HCV Infection.

*A documented negative HCV antibody laboratory test result followed within 6 months by a positive test (as described in the laboratory criteria for diagnosis) result does not require an acute clinical presentation to meet the surveillance case definition.

**Box 3. CDC/CSTE Chronic Hepatitis B Case Definition**

Clinical Criteria for Diagnosis

No symptoms are required. Persons with chronic hepatitis B virus (HBV) infection may have no evidence of liver disease or may have a spectrum of disease ranging from chronic hepatitis to cirrhosis or liver cancer.

Laboratory Criteria for Diagnosis

One or more of the following three criteria:

- Immunoglobulin M (IgM) antibodies to hepatitis B core antigen (IgM anti-HBc) negative AND a positive result on one of the following tests: hepatitis B surface antigen (HBsAg), hepatitis B e antigen (HBeAg), or nucleic acid test for hepatitis B virus DNA (including qualitative, quantitative and genotype testing), OR
- HBsAg positive or nucleic acid test for HBV DNA positive (including qualitative, quantitative and genotype testing) or HBeAg positive two times at least 6 months apart (Any combination of these tests performed 6 months apart is acceptable)

Probable Case Classification

A person with a single HBsAg positive or HBV DNA positive (including qualitative, quantitative and genotype testing) or HBeAg positive lab result and does not meet the case definition for acute hepatitis B.

Confirmed Case Classification

A person who meets either of the above laboratory criteria for diagnosis.

SOURCE: CDC/CSTE 2012 Case Definition for Chronic HBV Infection.

**Box 4. CDC/CSTE Chronic Hepatitis C Case Definition**

Clinical Criteria for Diagnosis

No symptoms are required. Persons with chronic hepatitis C virus (HCV) infection may have no evidence of liver disease or may have a spectrum of disease ranging from chronic hepatitis to cirrhosis or liver cancer.

Laboratory Criteria for Diagnosis

One or more of the following three criteria (except in persons less than 18 months of age, for whom only criteria 3 would meet the case classification criteria):

- Antibodies to hepatitis C virus (anti-HCV) screening-test-positive with a signal to cut-off ratio predictive of a true positive as determined for the particular assay as defined by CDC. (URL for the signal to cut-off ratios: https://www.cdc.gov/hepatitis/HCV/LabTesting.htm), OR
- Hepatitis C virus recombinant immunoblot assay (HCV RIBA) positive, OR
- Nucleic acid test (NAT) for HCV RNA positive (including qualitative, quantitative or genotype testing).

Probable Case Classification

A case that does not meet the case definition for acute hepatitis C, is anti-HCV positive (repeat reactive) by EIA, and has alanine aminotransferase (ALT or SGPT) values above the upper limit of normal, but the anti-HCV EIA result has not been verified by an additional more specific assay or the signal to cut-off ratio is unknown.

Confirmed Case Classification

A case that is laboratory confirmed and does not meet the case definition for acute hepatitis C

SOURCE: CDC/CSTE 2012 Case Definition for Chronic HCV Infection.

Underreporting of viral hepatitis infections has long been identified as a problem [11, 12, 13, 14]. Underreporting of these infections to state health departments and then to the Centers for Disease Control and Prevention (CDC) has complicated the understanding of the true viral hepatitis infectious disease burden. Estimation of viral hepatitis prevalence, incidence, and mortality has been complicated by the inability in most instances to correct for the bias caused by underreporting of these infections. Reporting of viral hepatitis infections has been severely limited by lack of resources and manpower at the local level necessary for evaluation of laboratory reports, and follow-up of reports to complete case reporting [1]. Such efforts are labor-intensive for public health department staff, as, for example, an average of four documents per case need to be evaluated to distinguish between acute, resolved, or chronic HCV case status [15]. CDC and state health departments rely on surveillance data collected and maintained by state health departments to inform strategies related to monitoring and tracking thousands of viral hepatitis infections each year.

The primary aim of the current study is to estimate the proportion of HBV and HCV infections that went unreported to the Pennsylvania Department of Health (PDoH), among patients in the Geisinger Health System of Pennsylvania. As a secondary objective, we study the association between underreporting of HBV and HCV infections to PDoH, and the select patient characteristics of interest: sex, age group, race/ethnicity, rural status, and year of initial diagnosis.

## Materials and methods

### Study design

**Fig 1:**
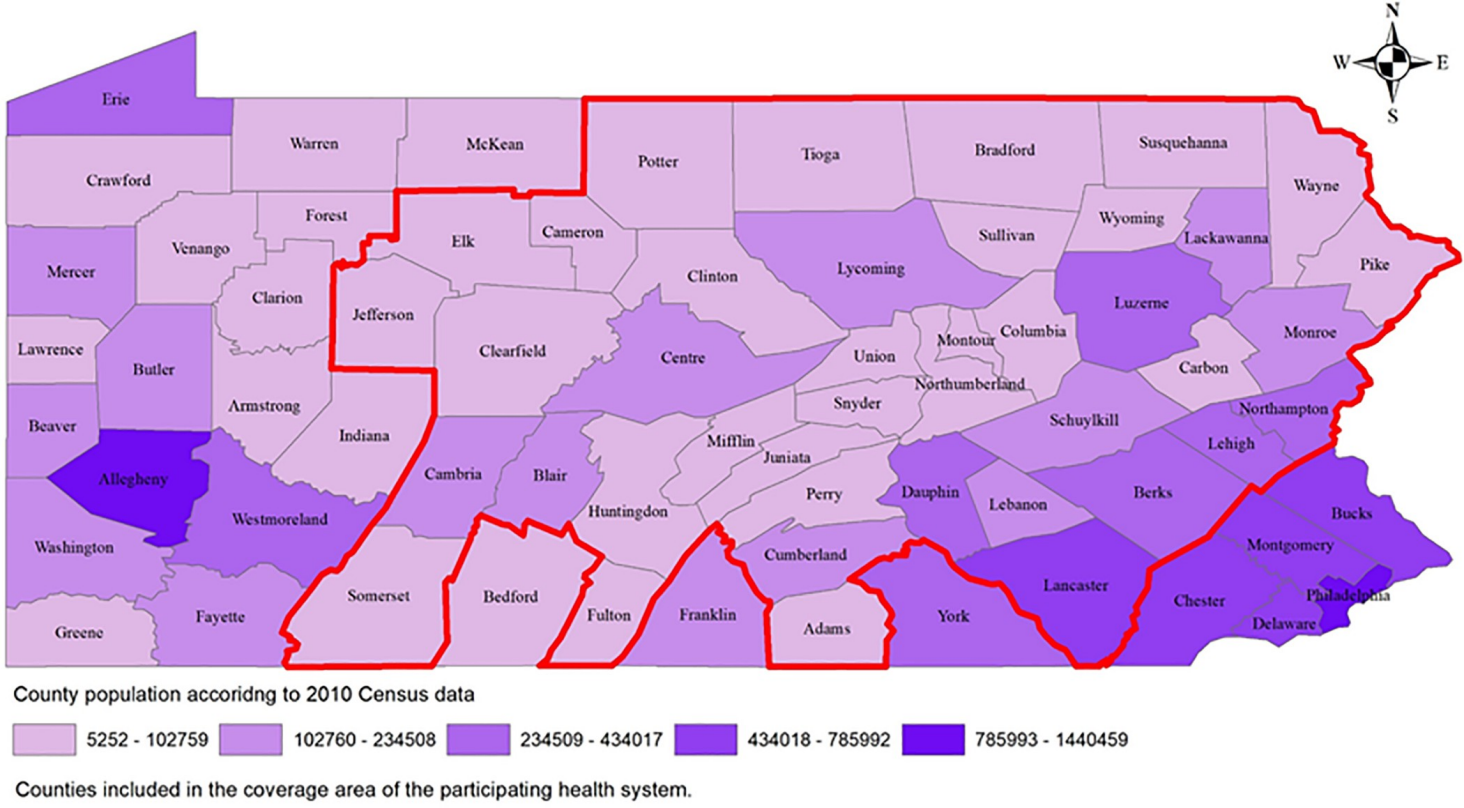
Geisinger Health System Coverage Area, Pennsylvania.

Geisinger Health System of Pennsylvania is a large integrated system that provides health services more than 3.0 million persons throughout 45 counties in central and northeastern Pennsylvania—about 20% of the state’s population, (see Fig 1) [16]. As part of the ongoing, multicenter Chronic Hepatitis Cohort Study (CHeCS), Geisinger Health System investigators compile data from patients diagnosed with chronic HBV or HCV infection.

Patients were included in the chronic hepatitis B and C cohorts based on fulfillment of a combination of laboratory-based and *International Classification of Diseases*, *Ninth Revision* (*ICD-9*)–based criteria. In brief, for inclusion in the hepatitis B cohort, patients had to fulfill at least 2 criteria (ie, 2 positive laboratory tests consistent with current HBV infection [positive for HBV surface antigen, e-antigen, or DNA test]), or a positive laboratory test and an *ICD-9* diagnosis code, or 2 *ICD-9* diagnosis codes obtained at least 6 months apart. Patients in all phases of chronic HBV infection (immune-tolerant, immune-active, and inactive or “immune carrier”) were included. For the hepatitis C cohort, a similar approach was used. The hepatitis C cohort was comprised of patients having ICD-9 diagnosis codes indicative of a hepatitis C infection, and either of the following: alanine aminotransferase elevation, a positive hepatitis C antibody test, or a positive hepatitis C RNA test, at least 6 months apart from the hepatitis C diagnosis date per the ICD9 classification. An expanded overview of the CHeCS inclusion criteria is shown in Box 5.

**Box 5. Criteria for identifying clinically confirmed cases of HBV and HCV infection in a cohort study– Pennsylvania, 2001–2015**

Confirmed chronic HBV infection if either of the following criteria are met*

Criteria 1

Specialist and primary-care provider documentation criteria

Written/dictated description in a progress note by a specialist (hepatologist, gastroenterologist, or infectious disease specialist) or patient’s primary-care provider† that describes patient as having chronic HBV infection, being a HBV carrier, or having nonreplicating HBV.

Criteria 2

Laboratory criteria

Any two of the following test results at least 6 months apart: HBsAg positive, HBV DNA positive, or HBeAg positive. (Any combination of these tests performed ≥6 months apart is acceptable.)

Confirmed chronic HCV infection if either of the following criteria are met§

Criteria 1

Specialist and primary-care provider documentation criteria

Written/dictated description in a progress note by a specialist (hepatologist, gastroenterologist, or infectious disease specialist) or patient’s primary-care provider† that describes patient as having chronic HCV infection.

Criteria 2

Laboratory criteria

Has any of the following test results:

- Anti-HCV (hepatitis C antibody) positive by enzyme immunoassay (EIA or ELISA)
- HCV RIBA (recombinant immunoblot assay) positive
- HCV RNA detectable
- Report of HCV genotype AND followed ≥6 months later by either of the following:
- HCV RNA detectable
- Report of HCV genotype

Criteria 3

Combination clinical and laboratory criteria

Patient has not presented with acute hepatitis (a discrete onset of any sign or symptom consistent with acute viral hepatitis [e.g., anorexia, abdominal discomfort, nausea, or vomiting, and either 1) jaundice or dark urine, or 2) serum ALT levels >400 IU/L]) AND has either of the following:

- HCV RNA detectable
- Report of HCV genotype

Source: Moorman AC, Gordon SC, Rupp LB, et al. Baseline characteristics and mortality among people in care for chronic viral hepatitis: the chronic hepatitis cohort study. Clin Infect Dis 2013; 56:40–50.

Abbreviations: HBV = hepatitis B virus; HCV = hepatitis C virus; HBsAg = hepatitis B surface antigen; HBeAg = hepatitis B e antigen; ICD-9 = International Classification of Diseases, Ninth Revision; EIA = enzyme immunoassay; ELISA = enzyme-linked immunosorbent assay; RIBA = recombinant immunoblot assay; ALT = alanine aminotransferase.

* Patients who are confirmed per these criteria to have had chronic HBV infection at any point, but who later cleared the disease (spontaneously or as a result of treatment), belong in the cohort and should be classified as having a confirmed case of HBV infection.

† This must be a textual description within a progress note, with or without an ICD-9 code. The primary-care provider should appear to have an informed, confident basis for the diagnosis based on serologic results and/or patient history, or the citation of outside laboratory studies that corroborate the diagnosis.

§ Patients who are confirmed via these criteria to have had chronic HCV infection, but who have been successfully treated and have cleared HCV RNA, belong in the cohort and should be classified as having a confirmed case of HCV infection.

The assessment of underreporting, was restricted to Geisinger Health System patients whom were residents of Pennsylvania, enrolled as CHeCS participants, and diagnosed with a chronic HBV or HCV infection between 2001 and 2015. Chronicity was established by trained abstractors, by a review of laboratory results and/or patient medical records to ascertain specific ICD9 codes (ICD 9—International Classification of Diseases, Ninth Revision) for HBV (070.22, 070.23, 070.32, 070.33, 070.2, 070.20, 070.21, 070.3, 070.30, 070.31, V02.61) or HCV (070.41, 070.44, 070.51, 070.54, 070.70, 070.71) [17].

### Dataset creation

In Pennsylvania, reporting of viral hepatitis B (HBV) and viral hepatitis C (HCV) infections to CDC has been mandated since 2002 [18]. Pennsylvania’s version of the National Electronic Disease Surveillance System (PA-NEDSS) has provided an electronic platform for streamlining disease reporting, and is used by Pennsylvania’s public health professionals for disease surveillance, outbreak detection, and case management [19]. Laboratory reports can be electronically transmitted or manually entered by local health departments, clinical providers, infection control personnel, or laboratories. PDoH began receiving electronic laboratory reports, during 2005. Once a HBV or HCV laboratory report is entered into PA-NEDSS, it is assigned to a public health expert for review. The reviewer conducts an investigation, consistent with the Pennsylvania viral hepatitis protocol for ascertaining if the infection meets the CDC/CSTE criteria for an acute/chronic HBV infection, or an acute/chronic HCV infection. PDoH employs several data quality strategies to ensure that duplicate laboratory reports are assigned to an unique state-assigned case identifier number.

In total, medical records for 2,533 study participants were merged with surveillance data records stored in PA-NEDSS. Data cleaning, standardization and extracting fields of interests were performed to prepare for matching. Data variables used in the matching process were the patient’s: first and last names; date of birth, zip code, street address, and city of residence. Levenshtein’s algorithm was used to identify identical matches between the two data systems [20]. Levenshtein’s algorithm (also called Edit-Distance) calculates the least number of edit operations (deletions, insertions, or replacement) that are necessary to modify one string to obtain another string. A matching score between 0 and 100 was created for each matched pair based on similarity. Non-identical matches deemed to be acceptable were those that had a matching score of 85 or greater.

### Dependent and independent variables (patient characteristic)

The patient’s case reporting status was the primary outcome variable of interest, and was characterized in one of two categories as follows: 1 = reported; 2 = not reported. Patient characteristics included in the analysis were: age group, gender, race/ethnicity, rural/urban status [21], and year of initial diagnosis. The variables were recoded from their native categories to new categories as follows: age group: (0–30 years, 31–44 years, 45–54 years, 55–64 years, and 65+ years); gender (female, male); race/ethnicity (Asian/PI NH, black NH, Hispanic, white NH); rural/urban status: (urban, rural); year of initial diagnosis (Prior to 1990, 1990–1999, 2000–2009, 2010–2015).

### Statistical analyses

With the prevalence ratio (95% confidence interval) as the measure of association between each patient and underreporting of viral hepatitis B and C infections in Pennsylvania, we used version 9.4 SAS-callable SUDAAN version 11 (RLOGIST procedure) to calculate the unadjusted proportion of chronic HBV or HCV infections diagnosed among Geisinger patients during 2000–2015, that were reported to PDoH as either an acute or chronic infection. We conducted a multivariable logistic regression analysis to study the association between each of the patient characteristics and the outcome variables {log-odds of HBV (HCV) reporting}. In the multiple logistic regression models fitted, independent covariates included patient characteristics with the following categories: Sex (male, female); Age-group (0–30, 31–44, 45–54, 55–64, ≥65 years); Race-ethnicity (black non-Hispanic, white non-Hispanic, Asian or Pacific Island non-Hispanic, Hispanic); Rural_Status (urban, rural); Initial_Diagnosis_Year (prior to 1990, 1990–1999, 2000–2009, 2010–2015). Reporting_status (reported, unreported) was the dependent variable in the multiple logistic regression models. We used the Wald Chi-square test to identify statistically significant prevalence ratios (p<0.05).

### Ethics statement

The CHeCS investigation follows the guidelines of the US Department of Health and Human Services regarding the protection of human subjects. The study protocol was approved and is renewed annually by an institutional review board approved by the Federal Office for Human Research Protections at each participating site.

## Results

Among the 2,533 Geisinger Health System study participants, 6.8% (n = 172) were living with HBV and 94.2% (n = 2,386) were living with HCV (Tables 1 and 2). Co-infections (patients infected with HBV and HCV) were present in 1.0% (n = 25) of these patients. Men accounted for 60.5% (104 of 172) of the HBV infections (Table 1) and 54.0% (1,289 of 2,386) of the HCV infections (Table 2). A disproportionate number of those infected with HBV were Asian/Pacific Islanders [17.4% or (30 of 172)], and a disproportionate number of those infected with HCV were non-Hispanic whites [89.9% or (2,144 of 2,386)]. It should be noted that Asian/Pacific Islanders and non-Hispanic whites represent 3.5% and 82.4% of Pennsylvania’s population, respectively [22].

**Table 1:** Percentage of viral hepatitis B infections reported to the pennsylvania department of health, 2001–2015.

Characteristic	PDoH	Geisinger	Unadjusted % Reported	Adjusted %Reported	Adjusted PR (95% CI)
Total	152	172	88.40%	82.60%	n/a
*Sex*					
Male	95	104	91.30%	86.10%	1.1 (1.0, 1.4)
Female	57	68	83.80%	76.20%	Ref
*Age*					
0–30	37	40	92.50%	88.60%	1.0 (0.8, 1.3)
31–44	55	63	87.30%	81.10%	1.0 (0.7, 1.3)
45–54	37	44	84.10%	74.90%	0.9 (0.7, 1.2)
55–64	13	14	92.90%	85.70%	1.0 (0.7, 1.4)
65+	10	11	90.90%	84.90%	Ref
*Race/Ethnicity*					
Asian/Pacific Islander	24	30	80.00%	75.20%	0.9 (0.7, 1.1)
Black, NH	18	19	94.70%	91.70%	1.1 (0.9, 1.3)
Hispanic	8	8	100.00%	n/a	n/a
WNH	94	106	88.70%	85.70%	Ref
*Rural Status*					
Urban	89	99	89.90%	84.00%	1.1 (0.9, 1.2)
Rural	63	73	86.30%	80.50%	Ref
*Year of Initial Diagnosis*					
Prior to 1990	11	11	100.00%	n/a	n/a
1990–1999	18	23	78.30%	77.70%	1.0 (0.7, 1.5)
2000–2009	107	119	89.90%	87.00%	1.1 (0.9, 1.5)
2010–2015	16	19	84.20%	76.60%	Ref

Abbreviations: PDoH = Pennsylvania Department of Health; PR = prevalence ratio; CI = confidence interval; NH = non-Hispanic; WNH = White, non-Hispanic

**Table 2:** Percentage of viral hepatitis C infections reported to the pennsylvania department of health, 2001–2015.

Characteristic	PDoH	Geisinger	Unadjusted % Reported	Adjusted % Reported	Adjusted PR (95% CI)
Total	2,257	2,386	94.60%	94.60%	n/a
*Sex*					
Male	1233	1289	95.70%	95.60%	1.03 (1.01, 1.05)
Female	1024	1097	93.30%	93.00%	Ref
*Age*					
0–30	525	544	96.50%	96.30%	1.17 (1.05, 1.30)
31–44	565	594	95.10%	95.10%	1.15 (1.02, 1.28)
45–54	825	868	95.00%	95.10%	1.15 (1.02, 1.28)
55–64	292	317	92.10%	91.40%	1.10 (0.98, 1.24)
65+	50	63	79.40%	85.10%	Ref
*Race/Ethnicity*					
Asian/Pacific Islander	4	7	57.10%	n/a%	n/a
Black, NH	136	144	94.40%	94.80%	1.00 (0.96, 1.04)
Hispanic	75	78	96.20%	65.00%	0.69 (0.33, 1.43)
WNH	2032	2144	94.80%	94.70%	Ref
*Rural Status*					
Urban	1149	1203	95.50%	95.60%	1.02 (1.00, 1.04)
Rural	1106	1181	93.60%	93.70%	Ref
*Year of Initial Diagnosis*					
2010–2015	306	311	98.40%	98.60%	1.08 (1.05, 1.12)
2000–2009	1534	1620	94.70%	94.60%	1.04 (1.00, 1.07)
1990–1999	330	364	90.70%	91.40%	Ref
Prior to 1990	87	91	95.60%	96.10%	1.05 (1.00, 1.11)

Abbreviations: PDoH = Pennsylvania Department of Health; PR = prevalence ratio; CI = confidence interval; NH = non-Hispanic; WNH = White, non-Hispanic

Geisinger Health System patients living with HBV were reported to PDoH, 88.4% (152 of 172) of the time (Table 1). The adjusted percentage of Geisinger Health System patients living with HBV that were reported to PDoH, was 82.6% (Table 1). Further, none of the select patient characteristics: gender, age group, race/ethnicity, rural-urban classification, or initial_diagnosis_year, were found to be statistically associated with a HBV infected patient’s infection going unreported to PDoH (Table 1).

Geisinger Health System patients living with HCV were reported to PDoH, 94.6% (2,257 of 2,386) of the time (Table 2). The adjusted percentage of Geisinger Health System patients living with HCV that were reported to PDoH, was also 94.6% (Table 2). Patients under the age of 31, had the highest reporting rate, with only 3.5% of their infections unaccounted for in the state registry (PA-NEDSS). Although HCV underreporting rates were minimal, when compared to adults < age 31 (adults whom were at least 65 years old), were 20% less likely to have their HCV infections reported to PDoH (prevalence ratio [PR], 1.2, [95% confidence interval [CI] 1.1, 1.3]) (Table 2). Underreporting of HCV infections, initially diagnosed during the 1990–1999 time period, was more problematic. Geisinger Health System patients, initially diagnosed during 1990–1999, were 8% less likely to have their HCV infections reported to PDoH, when compared to patients initially diagnosed during 2010–2015, (prevalence ratio [PR], 1.08, [95% confidence interval [CI] 1.04, 1.12]). (Table 2).

Geisinger Health System patients who were co-infected with both viruses (n = 25), were reported to PDoH, 72.0% (18 of 25) of the time. Male patients living with both viruses, were just as likely as female patients to go unreported to PDoH (P-value = 0.3408).

## Discussion

In the United States, all persons who test positive for HBV or HCV infections, should be reported to their state health departments to facilitate more precise measurements of the true burden of HBV and/or HCV infections. In Pennsylvania, all persons who test positive for HBV or HCV infections, must be reported to PDoH, cited in 28 Pa. Code § 27.21a. Prior to this study, very little was known about the extent that underreporting of HBV or HCV infections occurred in Pennsylvania. In this study, the assessment of underreporting was limited to Geisinger Health System patients whom were residents of Pennsylvania, enrolled as CHeCS participants, and diagnosed with a chronic HBV or HCV infection between 2001 and 2015. All of these patients should have been accounted for in the Pennsylvania viral hepatitis state registry (PA-NEDSS).

Based on the findings of this study, we concluded that 11.6% and 5.4% of the chronic HBV and HCV infections, went unreported to PDoH, respectively. This suggests that PDoH and public health providers in Pennsylvania were largely successful with registering HBV and HCV infections in PA-NEDSS, with respect to persons who tested positive for HBV or HCV infections. Findings from a similar CHeCS cohort of patients in Michigan, concluded that 18.0% and 35.0% of chronic HBV and/or HCV infections went unreported to the Michigan Department of Health, respectively [13]. The differences in the underreporting rates, may be due to the inherent differences in the state populations, and viral hepatitis surveillance practices. Underreporting was more substantial among patients that were living with both viruses, with 28.0% going unreported to PDoH. This suggests that PDoH and public health providers in Pennsylvania were less successful among persons who tested positive for both viruses. HBV and HCV co-infections are generally associated with increased risk of severe liver disease and liver cancer [23, 24, 25]. The lower reporting rate, among persons infected with both viruses, may be due to confusion surrounding how state investigators should have classified those infections. The CDC/CSTE case definitions do not address HBV and HCV coinfections.

As it pertains to secondary objective of this study: we concluded that the association between the select patient characteristics and underreporting of HBV infections to PDoH, was not statistically significant. However, underreporting of chronic HCV infections was more problematic among seniors (ages 65 and over), with 20.6% of the seniors living with HCV going unreported to PDoH. The Pennsylvania Hepatitis C Screening Act 87 (PA-HCV 87), enacted in 2016, will help decrease the underreporting of HCV infections to PDoH, among the baby boomer generation, persons born between 1945 and 1965 [26]. Subsequently, as Pennsylvania’s baby boomer population continue to age, PA-HCV 87 will also have a more impactful role in decreasing underreporting of seniors, ages 65 and over. The baby boomer population accounts for 75.0% of chronic viral hepatitis infections, in the United States [27]. Underreporting of chronic HCV infections was also problematic among Geisinger Health System patients initially diagnosed with a HCV infection during 1990–1999. CDC and CSTE have made substantial progress since the 1990s to standardize the viral hepatitis case definitions for viral hepatitis infections, and computerized reporting via the National Electronic Transmission Surveillance System (NETSS), and the National Electronic Disease Surveillance System (NEDSS) [28, 29]. The 1990s also correspond to a time-period when viral hepatitis infections were reported to state health departments largely by transmitting case reports via telephone faxes or US mail. The low reporting rates in the 1990s, may also reflect the inefficiency in reporting HBV and HCV infections during that time period. The increased utilization of electronic laboratory reporting systems (ELR), coincides with significant increases in funding to state health departments, via the CDC Epidemiology and Laboratory Capacity co-operative agreement, since 1995 [30].

This study has several limitations. First, the findings in this Pennsylvania based-study are promising, but do not account for underreporting among Pennsylvania’s general population that were not tested for HBV and/or HCV. Throughout the United States, the percentage of persons living HCV, who are among the undiagnosed and unaware of their infection status, may be as high as 42.0% - 50.0%; and the percentage of persons living HBV, who are undiagnosed and unaware of their infection status, may be as high as 20.0% - 31.0% [31, 32]. Second, these data are specific to a modern and well-integrated health care system and are therefore not generalizable to the entire state or nation. Reporting in a particular health system may not reflect reporting rates in other settings even within the same state. Third, some health system patients may have been diagnosed and reported in another state, despite current residence in the state of analysis. Fourth, our sample size was limited, particularly for the analysis of co-infections (n = 25), which may have prohibited us from fully exploring associations within the data.

The implications of this public health study, transcend the state of Pennsylvania. PDoH, Geisinger Health System of Pennsylvania, and the CDC were able to collaborate on this important study, by agreeing to share data and ideas. CDC and state health departments often collaborate to solve public health problems. The added dimension of collaborating with public health providers (hospitals, commercial laboratories, and insurance companies), will continue to help integrate ELR and EMR systems.
